# Dr. Power on the Female Economy

**Published:** 1821-09-01

**Authors:** 


*Essays on the Female Economy. 1. On the Periodical Discharge of the Human Female; with new Views of its Nature, Causes, and Influence on Disease; to which are added, Directions for its Management in the different Stages of Life. 2. On a Species of Abortion, not heretofore described, to which delicate Females in High Life are peculiarly liable; with a Mode of Treatment which has secured a happy Termination of the Pregnancy, where previously repeated Disappointment had been experienced.* By John Power, M. D. Physician-Accoucheur, &c. Octavo, pp. 101, with a plate. London, 1821.

In the *first* of these Essays Dr. Power attempts to unfold, upon natural and consistent principles, the nature of the "extraordinary periodical phenomenon which characterizes the human female," and which "has hitherto received no satisfactory elucidation." In the *second* Essay, his object is to call the attention of the profession to a species of abortion which he is disposed to believe "is now, for the first time pointed out."

"As this affection appears to be of not unfrequent occurrence, and, more particularly incidental to ladies in high life, to whom it may be a matter of the utmost importance to have children, although not exclusively confined to them; and as, when it has once taken place in any individual, it is liable to recur in all future pregnancies, it is hoped that the explanation which is attempted of its nature, and which appears to lead to a successful mode of practice, will not be deemed unacceptable to the profession, or useless to the public at large." Pref. iii.

Dr. Power observes, p. 8, that the *causes* of menstruation have been arranged under two heads—the efficient and *final* causes—the *latter* comprehending the *use* or purpose which it serves in the animal economy. We confess that we cannot conceive how the *use* of a thing can have any thing to do with its *cause*. We can readily imagine that the *use* of an apple is to make a tart and be eaten—but how tartification can be considered in the light of a final *cause* of the apple, we know not.*

It is much more easy to point out error than to establish truth; and therefore Dr. Power is successful enough in shewing that the MOON is not the cause of menstruation—nor redundancy of blood, local or general—nor a ferment in the uterus, as some have supposed.

Of the final *causes* (that is, purposes) of menstruation, the nutriment of the fœtus was one—and that, Dr. Power says, among the "more credible ones." It does not require much ingenuity to demolish this hypothesis, since, as Dr.P. obserses, "there do not appear any reservoirs in the womb, in which the fluid can be collected to answer this intention." How any physiologist could imagine that the fœtus was nourished by eating and drinking, *in utero*, it is unnecessary to inquire. It is not, perhaps, more absurd

* We do not accuse Dr. Power of any confusion of ideas on this head, since a quotation which he introduces a little farther on shews that Dr. Denman identifies *causes* and *uses*. "Had there been a gradual abatement in the (menstrual) discharge, in proportion to the increase of the fœtus, its *nourishment* might have been presumed to be one of the final *causes* of menstruation."—Denman. Here Dr. Denman evidently confounds *cause* and *object* together. Cause is defined by Locke to be "a substance exerting its power into act, to make one thing begin to be."

than some opinions now held orthodox, but which will afford many a hearty laugh to our great grand children.

It has been the more favourite opinion that menstruation serves to keep the female generative organs in a proper state for procreation. This, we think, is a very rational opinion. But Dr. Power says, if we admit it "we learn nothing in consequence." This may be the case, and how can we help it? The following passage might startle some sensitive old *maids*, if they were told that menstruation was the *effect* of the *generative process*.

"Nothing has been yet advanced to prove that it may not be a link in the *effects*, rather than the *causes* of the generative process; and, in short, that an improvement might be made upon the axiom, that "women who do not menstruate do not conceive," by substituting the following: a woman menstruates because she does *not* conceive." p. 8.

In short, if we apprehend the true bearing of Dr. Power's theory, it is this:—that were women in a state of nature they would begin to bear children at the period they now begin to mentruate—that during pregnancy and lactation, there would be no menses of course; and that, as soon as lactation was over, they would conceive again, and so on, through a long period of thirty or forty years.

"Were women living in a state of nature, is there not reason to infer that this discharge might be unknown? for supposing, (as that state would operate) that, between the age of 15 and 45, the female was employed in the constant task of renewing her species, one nine months would be employed in producing, and the next nine months in nourishing, her child, so that no time would be left for its occurrence." 11.

Heaven forbid that this state of nature—this "constant *task* of renewing the species," should ever come into vogue in this country; for if it did, New Holland, the Cape, and the plains of the Mississippi, would soon be too narrow for our overflowing population! It is but justice, however, to acknowledge the *fertility* of Dr. Power's imagination, in this new view of menstruation. We confess, indeed, that our author's reasonings are not so clear as to enable us to give any idea of them to our readers; but the following extract appears to contain the result of his theoretical views.

"In proportion as an ovum acquires a state of maturity in the ovarium, the uterus undergoes a correspondent preparation for its reception. Its vascular action is increased in a degree tending to a state of inflammation, and it wants but the additional stimulus of impregnation to determine it to the production of the deciduous secretion so requisite for its proper connexion with the ovum; if, however, the stimulus of impregnation is denied, this increased action is not carried to a sufficient height to produce properly that effect; nevertheless, it is sufficient to give rise to the effusion of a fluid, *which fluid is the menstrual fluid.* The secreting orifices of the uterine vessels being thus determined to an evacuation of their contents, this proceeds until, the ovarian action ceasing, the irritations of the uterine system are relieved, and the *effects* of the increased local action obviated.

"The quantity of fluid discharged during menstruation, and the difference in the qualities of that fluid from the decidua membrane, are not to be regarded as objections to this theory." 19.

The last paragraph in the above passage will give a very good idea of the facility with which authors can reconcile "objections to their theories." And, after all, we cannot help viewing the theory in question, as but a new version of that which ascribed menstruation to a local plethora of the uterine vessels—by which "the irritations of the uterine system are *relieved,* and the effects of *increased local action* obviated." In order, however, to do our author as much justice as our limits will allow, we shall lay before our readers a recapitulation of his theory in his own words.

"We shall now recapitulate, in a summary way, the opinions which have been above advanced.

"As the age of puberty approaches, and long before menstruation is established, the ovarian system of the human female, which had in earlier life lain inert, and almost invisible, begins to develope itself, and experiences that peculiar state of increased action, which determines it to the formation of an ovum, containing the female contribution to the embryo, and a store of nutritious matter for its support in the earlier periods of conception, before its full connexion with the uterus has enabled it to acquire supplies from sources external to itself.

"It is, then, necessary that, after receiving the vivifying principle of the male, this ovum should be received into a nidus called the uterus, through the medium of its connexion with which it derives from the maternal system the remaining nutriment and support, requisite for its full evolution and advancement to perfect animal life.

"During, and consentaneous with the above nisus of ovarian action, the uterus, by a correspondence with that action, is undergoing a state of preparation for the reception of the ovum into its cavity, and subsequent connexion with it. For this purpose, the uterus secretes a gelatinous fluid, resembling coagulable lymph, which inspissates, or concretes, into an organized substance, termed the decidua membrane; into this membrane the vessels of the ovum shoot, upon the common principle which effects the union of inflamed parts, and a connexion is established by the formation of a peculiar placental mass between the maternal and fœtal systems.

"Was the human female living in a state of nature, it is conceived that the above series of action would necessarily and invariably take place at the period of puberty. It happens, however, that in consequence of the operations of various physical and moral causes her impregnation is prevented, at that time, as well as at different subsequent periods of life. When this occurs, the actions of the generative system proceed, notwithstanding, as far as possible, in the discharge of their respective functions; but wanting the vivifying influence to be derived from the male, do not attain their full and proper acmé; the actions of the uterus in particular, except in certain cases of excessive local action, are insufficient for the formation of the decidua membrane; nevertheless, they are carried to a sufficient height to produce the effusion of a fluid from the secreting orifices; which fluid is the menstrual discharge.

"The uterine system is relieved by this discharge from its high state of local action, until the maturation and disappointment of another ovum, reproduces the phenomenon.

"Under this view of the subject, the efficient cause of menstruation may be defined—'An imperfect or disappointed action of the uterus in the formation of the membrane, (decidua,) which is requisite for its connexion with the impregnated ovum.'

"With regard to the final cause, it may be conceived that, looking upon the discharge as a deviation from what are to be considered the natural actions of the female, nature could have no final end in view, as it would be absurd to suppose an end contemplated, where no natural efficient cause was provided. Nevertheless, some anatomical evidence may be adduced, which proves that the efficient cause, whether natural or unnatural, was foreseen, if not intended. How, otherwise is the perforated hymen to be accounted for? the apperture of which appears evidently calculated for the transmission of the menstrual fluid." 28.

Towards the close of the first chapter, we find Dr. Power admitting the possibility of menstruation going on during utero-gestation, in opposition to Drs. Denman and Hamilton, who deny it. Of Dr. Power's mode of accounting for this phenomenon we shall take no notice, because we think it equally unsatisfactory as his theory of menstruation in general. But we may take this opportunity of stating that, we have had such ocular proofs of the occurrence in question, as to leave no more doubt in our minds of its occasionally happening, than we entertain of our existence at the present moment.

In the second chapter of the first Essay, we are happy to find Dr. Power on more practical grounds. This chapter is on the diseases of menstruation. The following short passage will shew that our author's reasonings about the *unnatural* phenomenon of menstruation dissolve before the light of clinical observation and experience.

"It appears, therefore, proper to regard the occurrence of menstruation under the present state of society, as a *proper, natural, and healthy secretion, tending to the sanity and welfare of the unimpregnated female system*, and every variation from its customary appearances, as a state of true deviation, and frequently of morbid derangement, since it is rarely found to take place, without being connected, more or less, with a disturbed state of the healthy functions." 36.

Dr. Power considers the deviations from the correct menstrual process under three heads, deficient, excessive, and irregular menstruation.

1. *Deficient Menstruation.* In order to keep up appearances of consistency, Dr. Power attributes the remote cause of this disordered state to "deficient action of the *ovaria*," the correspondent actions of the *uterus* being rendered proportionally defective. But he wisely adds a clause, that defective menstruation may be produced by "derangements in the whole or part of the general system."

This form of disease, when arising from imperfect organization, is, of course, incurable, and the patient is doomed to sterility.

"If, however, the organic defect exists only in a degree sufficient to produce a slow or retarded development of the ovum, it may be attempted to excite the ovarian actions to increased activity, by stimulating applications to the loins, thighs, or pubes; as frictions, stimulating plasters, or the electric fluid; or the semicupium might be used; or ligature or a tourniquet applied around the thighs; dancing and sexual excitements would probably be found beneficial; and lastly, some of those medicines which have been conceived to exert a specific action on the uterus; what these are is doubtful, but the intention, it is conceived, will most probably be answered by hellebore, savine, and madder." 39.

When the disease springs from chlorosis, the more prominent exciting causes, our author imagines, may be sought in too copious or rich and undigestible aliment affecting the alimentary canal, or a defect of proper nourishment, sedentary habits, &c. In the treatment of this form, after attending to the removal of causes;

"The digestive powers may be strengthened or confirmed by gentle stimulants, or by promoting the natural action of the alimentary canal, as by mild aperients, steel, bitters, myrrh, and aromatics. The local irritations acting on the uterus should be obviated, by carrying away, or preventing, accumulation in the intestinal canal, by the occasional use of the stronger purgatives, as the aloetic compounds, calomel, jalap, sulphas magnesiæ, &c. or by emollients and stimulating injections; and the natural actions of the ovarium may be excited or encouraged, in the manner before described." 41.

The same observations, Dr. Power thinks, will apply to *suppression* of the menses, after they have been once established.

In our author's remarks on menstruation *in excess*, we do not see any thing but what is familiar to every reader; we shall therefore pass on to the subject of *painful menstruation*, attended with the discharge of membranaceous substances, which our author seems to attribute to an inflammatory secretion of deciduous membrane, as believed by Dr. William Hunter.

> " The accompanying discharge is generally scanty, and not of the nature of menstrual fluid, but more resembling the effusions and weepings from wounded vessels, or the lochial discharge after parturition. The cause appears to be a too active or inflammatory state of the ovaria, throwing the corresponding actions of the uterus into excess; this may be the effect of peculiar constitution, influenced by constipation, luxurious living, or mental states." 50.

To relieve the existing paroxysm, Dr. Power recommends the spasmodic action to be taken off, by the free use of opium, or hyoscyamus, and the warm bath, with friction on the parts in pain. To these may be joined a cooling regimen, saline draughts, and occasionally antispasmodics. To prevent the return of these painful paroxysms, is a more important indication; and with this view, " irritation of the uterine system, from accumulation in the bowels and rectum, is particularly and regularly to be guarded against." The regimen should be cooling, and the local action of the uterine vessels may be diminished by the occasional application of leeches to the loins or pubes; while a counter-irritation may be kept up by a perpetual blister, or stimulating plaster to the back. We have seen the good effects of this plan in several cases of painful menstruation. In the sections on *irregular* menstrual actions we do not see any thing particularly requiring notice.

The 3d chapter of the work is on the "Management of Menstruation." This management consists in that attention to regimen and the state of the *chylopoetics* which is recommended by all ranks and classes of society, medical and not medical, yet attended to by few. Dr. Power repeats the usual tale of the injurious effects of *tea*, as a common beverage. For our own parts, we should be sorry to see this light and cheerful drink banished from our social meetings, even were the golden days of Queen Bess to return, and the ladies' breakfasts to consist once more of beef-steaks and "Coombe, Delafield, and Co." We agree with our author, that "three meals in the day are better than more;" nor will any but an Ascetic dispute the truth of the following position—"experience teaches us that, in human society, *dinner* is the best meal;"—at least it is by far the costliest. For habitual costiveness Dr. Power recommends the following pill, viz. aloes a scruple, ginger half a drachm, ipecacuan eight grains, divided into sixteen pills, of which one should be taken every day, an hour before dinner. We have lately seen two or three instances where a pill of the above description preserved its operative effect longer and more steadily than any other form that could be devised.

Dr. Power's directions respecting the management of the discharge, when it does take place, are not very much calculated for the professional reader. We shall therefore pass on to the second Essay.

*On a Species of Abortion.* After reading this Essay, we really could not find any particular species of abortion treated of; but only a new cause, (we believe, for it is dangerous to be positive about novelty) assigned for abortion occurring in the third, fourth, or fifth months of utero-gestation. This new cause is a *deficiency of oxygen,* owing to the chest being encroached on by the enlarging uterus.

> "In consequence of the difficulties opposed to the pulmonary actions of the mother, the absorption of the requisite quantity of oxygen to supply her own system, and the rapidly increasing demands of the child, is interfered with, so that one or both must necessarily fall into disease; as affecting the child, this is occasionally productive of its death; after which the actions of premature expulsion succeed to remove the dead mass from the uterus." 82.

This secret discovered, the remedies soon suggested themselves—pure air—drink acidulated with citric or nitric acids—and, if these fail, "oxigen gas is to be mixed with atmospheric air, and inhaled by means of an oiled silk bag." 91.

We shall proceed no farther. We are sorry that we cannot admire the novel theories introduced in this little work. We should be much better pleased to see Dr. Power detailing useful facts (of which his present situation must afford him abundance) than framing ingenious hypotheses. We respect this gentleman's talents, and we trust that the few criticisms in which we have indulged, may tend to direct those talents to better purposes than they are here expended on.

